# Temperature-Dependent Pupation Depth in the Oriental Fruit Fly *Bactrocera dorsalis* and Its Implications for Biological Control

**DOI:** 10.3390/insects15110873

**Published:** 2024-11-06

**Authors:** Mu-Rung Lin, Toshinori Okuyama

**Affiliations:** Department of Entomology, National Taiwan University, Taipei 10617, Taiwan

**Keywords:** host–parasitoid interactions, Tephritidae, pupation depth, temperature, soil

## Abstract

The oriental fruit fly, *Bactrocera dorsalis* (Hendel) (Diptera: Tephritidae), is a notable agricultural pest that undergoes pupation in the soil. Mortality risk from predation and parasitism decreases as the depth of the pupal location increases from the ground surface, with a one-centimetre increase in depth causing a significant change. Soil properties, such as moisture and hardness, influence pupation depth, but the effect of temperature has not been fully tested. This laboratory study examined whether a biologically important variation in pupation depth (e.g., one centimetre) is caused by naturally experienced temperature variations (20 to 35 °C) in *B. dorsalis*. The temperature–pupation depth relationship revealed a unimodal pattern, with the deepest pupation occurring at intermediate temperature levels and shallower pupation at the two extreme temperature ranges. Strong quantitative effects were observed, with the highest mean pupation depth of 40.8 mm at 27.5 °C and the lowest mean pupation depth of 15 mm at 35 °C. The observed quantitative effect suggests that temperature can strongly affect pupal mortality from predators and parasitoids by influencing pupation depth. Future studies that reveal the ability of biological control agents to forage underground for pupae at different temperatures are awaited, as this is key information for evaluating the effectiveness of these agents.

## Simple Summary

The oriental fruit fly, *Bactrocera dorsalis*, pupates in the soil, and the depth at which it pupates influences its susceptibility to natural enemies, which plays a key role in biological control programs. This laboratory study examined the relationship between temperature and pupation depth, showing that even small changes in temperature lead to significant variations in pupation depth. This result suggests that temperature may affect interactions between *B. dorsalis* and its natural enemies in ways that were previously overlooked.

## 1. Introduction

The oriental fruit fly, *Bactrocera dorsalis* (Hendel) (Diptera: Tephritidae), is an internationally recognised serious agricultural pest. *Bactrocera dorsalis* is currently globally distributed, including South and North America, Africa, Oceania, and Europe [1]. *Bactrocera dorsalis* can infest hundreds of plant taxa, including 79 plant families with infestation records under natural field conditions and an additional 51 families without validated records [2]. Phytosanitary treatments against *B. dorsalis* are required for the international trading of numerous host crops, including papaya, mango, dragon fruit, and lychee [3]. Adults are free-living, making them the main target of various control measures such as the use of chemical insecticides [4], male annihilation [5], and bait application [6]. Dealing with immature stages, on the other hand, poses a greater difficulty because they are physically buffered from the external environment; eggs and larvae develop in fruits, and pupation takes place in the soil. Biological control agents, such as parasitoids and predators, however, are capable of attacking immature stages of *B. dorsalis* [1], and the integration of biological control into a pest management program is thought to be a desirable approach for managing this pest.

The pupation depth of *B. dorsalis* is an important factor when examining the interaction between the pupal stage of *B. dorsalis* and its natural enemies. Many species of tephritid flies, such as *B. cucurbitae*, *Ceratitis capitata*, and *Rhagoletis mendax*, among others, pupate in the soil [7,8,9,10]. Therefore, the consideration of pupation depth is important when managing the populations of tephritid flies, not restricted to *B. dorsalis*. Pupae of tephritid flies are attacked by various predators (e.g., ants, ground beetles, birds) and parasitoid wasps [11,12,13,14,15,16]. The mortality of pupae caused by predators and parasitoids generally decreases as pupation depth increases [12,17,18,19,20], which is expected because most predators and parasitoids live above ground, and pupae located near the ground surface are inevitably more likely to be encountered first. In addition to the qualitative effect, the strong quantitative effect of pupation depth on mortality must be explicitly considered. For example, the parasitism of *Spalangia endius* (Walker) (Hymenoptera: Pteromalidae) on *B. cucurbitae* decreased from approximately 80% to 30% when pupation depth increased from 1 cm to 2 cm [19]. The pupation depth of tephritid flies naturally varies by several centimetres [21], and any factors affecting pupation depths can have substantial impacts on the efficacy of natural enemies when used as biological control agents.

Various factors influence the pupation depth of tephritid flies. From a fly’s perspective, pupating at a greater depth offers the benefit of reduced mortality risk from natural enemies, as discussed above, but it also entails lower odds of successful emergence (i.e., eclosed adults in the soil may fail to reach above ground) [22]. In *B. dorsalis*, the average pupation depth increases with the number of concurrently pupating larvae [17]. This density-dependent pupation depth may be partially explained by the density-dependent emergence success of eclosed adult flies, as the emergence success rate is higher when adults synchronously eclose in the soil [23]. Physical characteristics of soil, such as moisture and compaction, affect pupation depth [8,9,10,24,25], presumably by directly affecting how easily larvae can manoeuvre within the soil. On the other hand, the impact of certain general physical factors, such as a moderate range of temperatures, which may not strongly affect soil hardness and texture, has been minimally studied in the olive fly, *B. olea*. Previous research has indicated that the effect of temperature on the median pupation depth is weak, with a difference of less than 1 cm between 10 °C and 30 °C [26].

In host–parasitoid population models, the parasitism risk experienced by hosts is a key parameter that affects the stability of the equilibrium, as well as the equilibrium densities of the species [27,28]. In other words, temperature may be a key factor that influences population dynamics by affecting parasitism risk mediated by pupation depth. The population dynamics of *B. dorsalis* and other tephritid flies can vary substantially, not only between year [29,30] but also within a season (early vs. late summer) [31]. Changes in parasitism risk caused by temperature variability within and between years may be a contributing factor, although temporal changes in pupation depth in the field have not been studied.

This descriptive study (as opposed to a hypothesis-driven study) aims to delineate the relationship between temperature and pupation depth in *B. dorsalis*, as this relationship is crucial for understanding the interactions between *B. dorsalis* and its natural enemies. A temperature range of 20 °C to 35 °C, which reflects typical fluctuations during the agricultural season in subtropical regions, was considered particularly relevant to the fruit-harvesting period. Although fruits may also be harvested in cooler seasons, the population size of *B. dorsalis* is low in cold months [29], and low temperatures also strongly affect the performance of parasitoids. For example, *Diachasmimorpha longicaudata* (Hymenoptera: Braconidae), an important agent of tephritid flies, cannot develop successfully at 15 °C [32]. The main focus is whether a difference in pupation depth of a centimetre or more (i.e., a biologically meaningful difference) is observed within this realistic temperature range for biological control. Additionally, to address the effect of soil drying over time as a confounding factor, the pupation rate was also examined.

## 2. Materials and Methods

### 2.1. Insects

Cultures of *B. dorsalis* were maintained in the laboratory. *Bactrocera dorsalis* larvae were raised on an artificial diet consisting of wheat bran, sugar, yeast, citric acid, and water, in a weight ratio of 25:10:5:1:50. To prevent the development of fungus, a mixture of sorbic acid (0.8 g) and nipagin (0.8 g) dissolved in 1 mL of 70% ethanol was added to 180 g of the artificial diet. Adult flies were provided with a mixture of sucrose, yeast powder, and peptone in a ratio of 6:1:1, along with solid sugar and water, which were always available. Flies laid eggs into portion cups containing the artificial diet when the cups had holes made with needles. Larvae developed inside the artificial diet until pupation (further described below). Pupae were then moved to adult fly cages to maintain the adult populations. The species has been maintained in a room with a controlled temperature of 28 °C since 2022. Sunlight was available through the room’s windows.

### 2.2. Pupation Depth

The pupation depth of oriental fruit fly larvae was examined at seven temperature levels, ranging from 20 °C to 35 °C with an increment of 2.5 °C. The experimental arena was a plastic cup (height: 110 mm, bottom diameter: 94 mm, top diameter: 110 mm) filled with peat moss (Sinon Corporation, Taichung, Taiwan). The peat moss was stored at the respective temperature in a sealed bag for a week before being used in the experiment. The relative humidity of the peat moss used in the experiment was measured with a soil humidity tester (Model: YY-1000, Shen Zhen Yage Technology Co., Shenzhen, China) immediately before being used in an experimental trial.

The experiment was conducted in temperature-controlled growth chambers under constant darkness (CK-68EX, Chang Kuang, Taipei, Taiwan). Constant darkness was used to eliminate light access from inside the soil. For example, if larvae are at the wall of a plastic cup, they are still exposed to light even when they are deep in the soil, which would not occur in the field. A cup (height: 48 mm, diameter: 60 mm) filled with the artificial diet containing *B. dorsalis* eggs was placed in a larger cup (height: 78 mm, diameter: 96 mm). When third instar larvae developed on the artificial diet were ready to pupate, they emerged from the cup containing the artificial diet and dropped into the bottom of the outer cup. Larvae in the outer cup were used in the experiment. Larvae continuously move from the diet cup to the outer cup. At the start of the experiments, larvae and pupae already located in the outer cup were removed, and only newly emerged larvae were used. The duration that larvae stayed in the outer cup was less than 30 min, as this was the maximum time needed to prepare the experimental setups. The experimental arena (described above) was filled with 8 cm of peat moss without applying any pressure from above. One larva was placed at the centre of the peat moss surface and kept in the growth chamber. Pupation depth was determined by gradually removing a small amount of peat moss using a spatula. A thin stick was inserted into the peat moss such that its end touched the bottom of the cup, and the surface level of the peat moss was marked on the stick. When a pupa was found during excavation, its location was marked on the stick, and the distance from the surface mark to the pupa mark was recorded as the pupation depth. The lengths of pupae were recorded with a caliper. The number of replications was 46 (20 °C), 46 (22.5 °C), 36 (25 °C), 38 (27.5 °C), 41 (30 °C), 41 (32.5 °C), and 44 (35 °C). Three growth chambers were used. Initially, 20 °C, 25 °C, and 30 °C were tested, followed by 22.5 °C, 27.5 °C, and 35 °C. Lastly, 32.5 °C was tested.

### 2.3. Pupation Rate

This study aimed to examine the effect of temperature on pupation depth independent of soil characteristics; thus, the moisture level was standardised, as described above. However, temperature can also impact the moisture level of peat moss through evaporation over time, particularly if the larvae used in the experiment did not pupate for an extended duration. An experiment was conducted to determine how quickly larvae metamorphose into pupae at the seven temperature levels.

The experimental procedure was largely the same as the pupation depth experiment described above. One difference is that a small cup (height: 48 mm, diameter: 60 mm) was used as the pupation environment. Peat moss was filled to a depth of 4 cm, and a larva was placed at the centre of the peat moss surface. The determination of whether or not a larva became a pupa was examined at 2, 4, 6, and 24 h after its introduction. For each temperature and duration combination, 30 replications were performed.

### 2.4. Data Analysis

The differences in mean soil humidity between temperature levels were analysed using ANOVA. The differences in mean pupal length between temperature levels were analysed using Welch’s ANOVA due to significant deviations from homoscedasticity, as tested by Levene’s test. No significant deviation from normality was observed for soil humidity across all temperature levels, but significant deviations were observed for pupal length at two temperature levels (20 °C and 27.5 °C). Since it is not appropriate to apply transformations to only two groups, the raw (untransformed) data were used for the analysis. The relationship between pupation depth and temperature was described using a Gaussian nonlinear regression model, where the average pupation depth was modelled as $ax\left(x-x_{0}\right){\left(x_{1}-x\right)}^{1/m}$, with x representing temperature and a, $x_{0}$, $x_{1}$, and m as model parameters [33]. To test for a significant effect of temperature, this model, incorporating the temperature effect, was compared to a model with a constant pupation depth across temperatures using AIC. The AIC values with and without the temperature effect, respectively, are denoted as AIC_1_ and AIC_0_. Models with smaller AIC values provide a better description of the data. When AIC₀ – AIC₁ > 2, temperature is considered to have a significant effect [34]. In the pupation rate experiment, the proportion of larvae that pupated was described by a binomial generalised linear model, and potential differences among temperature levels were tested by a multiple comparison test using Tukey contrasts.

## 3. Results

**Figure 1:**
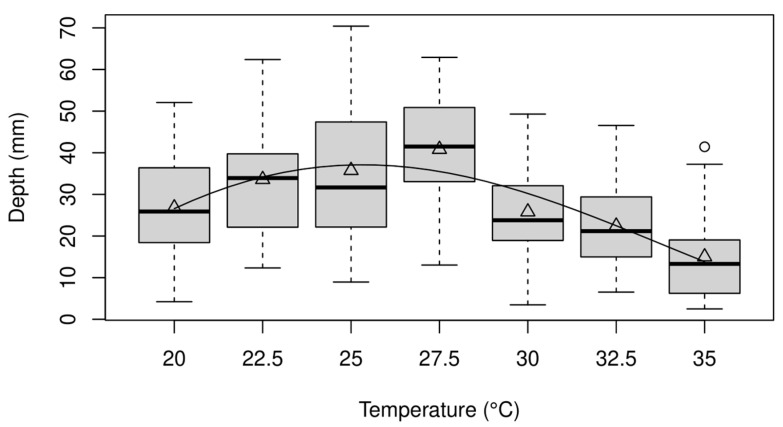
Relationship between temperature and pupation depth in box plots. The horizontal line and triangle inside the box represent the median and mean, respectively. Data values outside 1.5 times the interquartile range are shown as circles. The line represents the mean pupation depth model $ax\left(x-x_{0}\right){\left(x_{1}-x\right)}^{1/m}$, with estimated parameters provided in the main text.

The relationship between temperature and pupation depth exhibited a unimodal shape (Figure 1). The maximum likelihood estimates for the model of average pupation depth are as follows: a = 0.0002, $x_{0}$ = 15, $x_{1}$ = 42.1, m = 0.4, and the standard deviation of the Gaussian model is σ = 12.4. The deepest average temperature occurs at 25.3 °C, according to the model. The AIC values, AIC_1_ and AIC_0_, are 2311 and 2397, respectively, indicating a significant effect of temperature on pupation depth. The average ± SD (mm) of pupal lengths were 4.64 ± 0.42 (20 °C), 4.73 ± 0.43 (22.5 °C), 4.71 ± 0.35 (25 °C), 4.76 ± 0.26 (27.5 °C), 4.65 ± 0.38 (30 °C), 4.84 ± 0.30 (32.5 °C), and 4.73 ± 0.31 (35 °C), and were not different among temperature levels (Welch’s ANOVA, *F*_6,125.71_ = 1.66, *p* = 0.137). The variance of pupation depth differed among temperature levels (Levene’s test, *F*_6,285_ = 2.54, *p* = 0.02).

**Figure 2:**
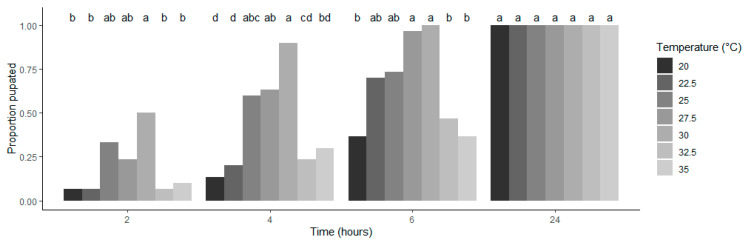
Relationship between the proportion of larvae pupated and the duration since the larvae were placed on peat moss. Groups with the same alphabet are not significantly different at α = 0.05 (multiple comparison procedure using Tukey contrasts).

Temperature influenced the rate of pupation (Figure 2). The proportion of pupated individuals was equivalent at intermediate temperature levels (between 25 and 30 °C within 4 h, and between 22.5 and 30 °C within 6 h) (Tukey multiple comparisons, *p* > 0.05) and was higher than that at other temperature levels (Tukey multiple comparisons, *p* < 0.05). The differences in pupation rates among temperatures disappeared at 24 h because all individuals pupated within 24 h regardless of temperature. The average ± SD of relative humidity (%) of peat moss used in the experiment at the seven temperature levels were 11.8 ± 0.69 (20 °C), 12.05 ± 0.83 (22.5 °C), 11.95 ± 0.81 (25 °C), 12.12 ± 1.02 (27.5 °C), 12.33 ± 1.00 (30 °C), 12.04 ± 0.75 (32.5 °C), and 12.10 ± 0.88 (35 °C) and were not different among temperature levels (ANOVA, *F*_6,285_ = 1.535, *p* = 0.167). The absence of initial humidity differences between temperature levels and the fast partition rate minimises concerns about temperature-dependent water evaporation affecting the observed pupation depth.

## 4. Discussion

Pupation depth is crucial for understanding the relationship between *B. dorsalis* pupae and their natural enemies. This study not only confirms that temperature affects pupation depth, which was previously unknown, but also highlights that the effect is significant enough to warrant attention when developing biological control programs. A temperature difference as small as 2.5 °C (e.g., between 27.5 °C and 30 °C) led to a change in pupation depth of more than one centimetre, which can greatly affect the mortality risk of pupae, as discussed in the introduction. Since parasitoids are the primary biological control agents targeting *B. dorsalis* pupae, the implications of temperature-dependent pupation depth on host–parasitoid interactions are discussed below. However, many of these considerations also apply to other natural enemies, such as predators.

Both the mean and variance of pupation depth were influenced by temperature (Figure 1). Variability in pupation depth contributes to variability in parasitism risk, a key factor determining the dynamics of host–parasitoid populations [27,35,36]. In conventional host–parasitoid studies, variability in parasitism risk was mainly assumed to come from variability in parasitoid density among patches [27,37], but the present study shows that this crucial parameter is temperature-dependent and dynamically changes at a single location. Another important detail is that experimental studies show parasitoids are incapable of parasitising hosts that are located sufficiently deep. For example, *S. endius* cannot parasitise hosts located at 5 cm below the ground surface [19]. Therefore, pupae located at sufficient depths are considered to occupy refuges, which is another key factor that affects host–parasitoid population dynamics [28,38]. Previous studies that examined the effect of temperature mainly focused on its effect on the demographic parameters (e.g., development rates and fecundity) of insects [39,40,41], but the results of the present study suggest an important and novel role of temperature in host–parasitoid interactions.

Because the experiment was conducted in an artificial setup, the results should be interpreted with appropriate caution. For example, since pupation depth is influenced by soil properties, the results of this study are specific to the soil used in the experiment (i.e., soft peat moss with a moderate level of moisture). If the substrate used in the experiment were harder for larvae to burrow into (e.g., due to differences in soil texture), pupation depth would generally be shallower. Additionally, temperature was held constant, although temperature is variable in the field. However, since soil temperature is more stable than air temperature [42] and pupation occurred over a relatively short period (Figure 2), the effect of temperature variability on pupation depth may be less significant than its effect on other life history parameters of insects [43,44]. The fast pupation rate was also used to justify that soil moisture differences had a minimal effect in this experiment. However, temperature-dependent evaporation of water from the soil substrate, if it occurred, is not necessarily unrealistic and is expected to happen in the field, although soil moisture is strongly influenced by weather events, regardless of temperature.

The effectiveness of parasitoids as biological control agents depends on pupation depth as well as the ability of parasitoids to forage in the soil. However, the latter information is largely unknown. As discussed above, studies indicating the pupation depth-dependent parasitism rate exist, but we do not have essential information such as which parasitoid species has a better ability to forge in the soil. In fact, the parasitism behaviour of pupal parasitoids of tephritid flies, such as *Dirhinus giffardii*, *D. longicaudata*, and *S. endius*, have commonly been studied by providing pupae directly exposed to parasitoids [45,46,47,48]. However, species that effectively parasitise exposed hosts (e.g., hosts on the ground surface) may not perform well with underground hosts and vice versa. Furthermore, the effectiveness of parasitoids in parasitising underground hosts depends on the soil, which is a complex mixture of substances. Two soils that share similar properties in certain factors (texture, moisture, pH, compactness, etc.) may still differ in other aspects. Therefore, the quantitative comparison of parasitoid performance between two independent studies may be unreliable (the same can be said for quantitatively comparing the pupation depth of flies among different studies, as discussed above). When comparing parasitoids regarding their ability to parasitise underground hosts, multiple parasitoid species should be tested with the same soil within the same study. The selection of biological control agents should be based on the direct comparison of candidate species under the specific soil found in the agricultural field where the species are planned to be released.

This study did not consider factors relating to the adaptation of *B. dorsalis*, but it is an important factor in the interpretation of the results. The *B. dorsalis* culture was maintained at 28 °C before being used in the experiment. Therefore, it may be reasonable to consider that the flies were adapted to the temperature. If the temperature suddenly changes (as in this study), the effect of temperature change is expected to follow the observed pattern (Figure 1). In other words, if *B. dorsalis* was adapted at a higher temperature to begin with (e.g., maintained at > 30 °C), their performance at a higher temperature may be different from what is observed in this study. In fact, although some individuals survived at 35 °C in this and previous studies [41,49], other studies reported the survival rate of *B. dorsalis* at 35 °C is zero [39,40]. Such a difference is expected to result from pre-existing genetic differences in tolerance to high temperatures. Because temperature-dependent performance is expected to be at least partially heritable [50], any descriptive studies on the effect of temperature have to be carefully interpreted with this detail. To better understand how a change in temperature affects the interaction between *B. dorsalis* and its natural enemies, we must explicitly consider not only how *B. dorsal* adapts to the change in temperature but also how its natural enemies evolve in response to temperature variations [51], especially when considering the effect of a long-term, gradual change in temperature such as global warming.

Our findings highlight the importance of temperature as a key factor influencing the pupation depth of *B. dorsalis*. The effect of temperature on pupation depth described in this study is qualitatively similar to the aforementioned pattern in *B. oleae* [26], but greater quantitative effects were observed in *B. dorsalis* (this study). However, as discussed above, such a quantitative comparison may not be reliable due to soil differences. Future research that evaluates the temperature-dependent foraging success of pupal parasitoids, explicitly considering pupae at various soil depths, will be valuable for determining the appropriate species for particular applications, as there is currently no information available to compare parasitoid species regarding this detail. Temperature is a dynamic factor operating on both fast (e.g., seasonal changes or daily variation) and slow time scales (e.g., climate changes over the years). The impact of temperature-dependent pupation depth is crucial not only for the consideration of biological control agents but also for understanding the dynamics of host–parasitoid populations beyond agricultural systems.

## Data Availability

The data supporting the findings of this study are available from the corresponding author upon reasonable request.
